# How Bioethicists Can Contribute to Democratic Deliberation

**DOI:** 10.1111/bioe.70168

**Published:** 2026-09-11

**Authors:** Ben Saunders

**Affiliations:** ^1^ University of Southampton Southampton United Kingdom of Great Britain and Northern Ireland

## Abstract

It is sometimes suggested that policymaking on bioethical questions should be more democratic, either through direct popular participation (as in a referendum) or through improved forms of representation (such as random mini‐publics or elected officials). Such proposals seem to leave no special place for input from bioethicists. This paper argues that it is not contrary to democracy to give bioethicists additional influence over relevant policy areas. First, while referenda, mini‐publics, or electoral representation may be more democratic in some respects, these processes also have limitations, in particular excluding the interests of disenfranchised groups (such as children or future generations). Second, I suggest that it is wrong to think of democracy only in terms of something like the will of the citizens. Democracies usually involve mechanisms to divide power, potentially blocking a majority's will to protect minority rights. Bioethicists may serve this function, particularly when they raise objections on behalf of minorities or disenfranchised groups.

Some bioethical questions concern decisions of private individuals (or close family units). For instance, individuals deciding whether and how to reproduce, get themselves (or their children) vaccinated, register as an organ donors and so forth. But others concern collective matters, such as state regulation of these practices. For instance, whether and on what terms the state should permit abortion, reproductive technologies (such as IVF or sex‐selection), vaccine refusals, organ donation and so forth. While these questions of the latter sort raise different ethical issues, what they have in common is that they are questions of public policy, rather than personal morality.^1^ That is, they are concerned with what the state should or should not do, exercising its coercive power. Recognition of this has led to a ‘political turn’ in bioethics.^2^ Many authors have suggested that questions concerning state action should be resolved through political means, preferably via some form of democratic process.^3^ This is often taken to require more public participation in policymaking. However, this raises questions about the influence of bioethicists.^4^

Democracy means rule by the people. It is usually understood to require (near) universal inclusion and equal power. This can be contrasted with what is sometimes called ‘epistocracy’ (rule by the knowledgeable), which may involve either restricting power to those deemed knowledgeable or giving them greater power, for instance through plural voting.^5^ However, these epistocratic systems face many objections.^6^ In particular, the ‘demographic objection’ is that knowledgeable groups are generally unrepresentative of the wider population and this may bias their decision‐making [14, ch. 11] This objection against epistocracy would seem to apply to proposals that give special influence to bioethicists, since they are also an unrepresentative group [15].^7^ If bioethicists are, for instance, disproportionately white, then this may distort their views, leading them to neglect issues of racism [16].

Despite these concerns, this paper defends a potential role for bioethicists in policymaking. While I do not reject claims that ordinary citizens should have *some* involvement, perhaps even more than they currently do, I resist the idea that democracy should deny any role to expertise, on grounds of political equality. That is, I defend the *permissibility* of giving bioethicists privileged influence, against accusations that this is undemocratic. However, I do not argue for the stronger claim that bioethicists should or must be given special privileges in policymaking.

It is not my goal to make specific institutional recommendations, because these are likely to depend on context. However, I argue that bioethicists can legitimately be given a formal role in the policymaking process.^8^ This may be through open consultation or involve a select group, such as a presidential commission.^9^ Either way, their role need not be a ‘decision‐making’ one. It may be primarily deliberative, for instance offering expert commentary on draft legislation or bioethical issues. Alternatively, they could draft policy proposals, for ratification by others.^10^ Either way, they would not actually make, or have vetoes over, policy. Nonetheless, they would have privileged input into shaping decisions. This might offend against certain understandings of democracy, which emphasise equality and popular will, but I argue these are simplistic. It is consistent with other, richer understandings of democracy, which often include various mechanisms to protect rights from popular control.^11^

I acknowledge the concern that bioethicists are often *descriptively* unrepresentative, that is, unlike the wider population in various respects (such as sex, race, religiosity).^12^ There is a danger that this may bias their views. However, supposedly ‘more democratic’ alternatives have problems of inclusion and representation too. A popular referendum involves mass participation, but this need not be informed and, in any case, can only reflect the views of those who participate. The use of randomly selected mini‐publics can overcome some of these deficiencies, but there is still a danger that those involved are either uninformed or excessively influenced by experts and advisers. Further, there are also concerns about how representative these mini‐publics really are, since some groups may refuse to serve while others (e.g., children or foreigners) may not even be eligible for inclusion.^13^ The more familiar alternative is *electoral* representation, which is supposed to give representatives self‐interested incentives to serve the interests of their constituents. However, elections are a rather crude accountability mechanism though and, again, only promote responsiveness to those who are enfranchised (and perhaps only those who actually vote). Thus, each of these supposedly ‘more democratic’ options may also fail to protect certain vulnerable interests, particularly of non‐enfranchised groups. Bioethicists, in contrast, may be well‐placed to voice objections to policy proposals and, in doing so, may even speak on behalf of those who have no voice of their own.

To be clear, none of this means that we should abandon the use of popular referenda, mini‐publics, or elected representatives. These things have their uses. Further, used in combination, each may help to correct deficiencies of the others. But, in this spirit of pluralism, we might want to supplement them with other institutions too [18]. This is where bioethicists may be useful. They can speak on behalf of groups or interests that may be neglected by seemingly more democratic participatory procedures. In doing this, they may sometimes be acting against a popular majority, but that does not mean they are acting undemocratically.

## 1 Concerns About Bioethical Expertise

The suggestion that bioethicists should occupy a privileged position when making policy on bioethical issues appears undemocratic. Even those recent writers who endorse technocratic decision‐making in other areas often balk at the idea of applying this logic to moral questions.^14^ One particular cause for concern about entrusting decisions to bioethicists is that they are, as a group, unrepresentative of the wider population.^15^ This lack of descriptive representativeness may lead them to favour certain views or interests, neglecting marginalised perspectives (the demographic objection). For instance, if they are generally socially privileged, then they may not be attuned to the concerns of disadvantaged and marginalised groups around topics such as assisted dying [20, pp. 641–2].

While this is a serious worry, there are several things to say in response. First, while bioethicists may under‐represent some groups/views, we should acknowledge that they are far from homogeneous. Bioethics is an inter‐disciplinary field, so different practitioners often bring diverse perspectives.^16^ Further, bioethicists have increasingly drawn on a wide range of intellectual traditions, including non‐Western ones.^17^ Second, the survey data about bioethicists concerns the group as a whole.^18^ But a particular group of bioethicists involved in the policy process need not be representative of bioethicists as a whole. If selecting a panel or committee of bioethicists, one could deliberately over‐sample certain groups (e.g., racial minorities or religious believers) who are under‐represented amongst bioethicists, to make the panel more representative (descriptively) of the wider population.^19^

Third, it might be that what matters is the representation of certain viewpoints or discourses, regardless of who expresses them [25]. It may not be important how many deliberators hold these views, so long as they get a fair hearing. One skilled advocate may be enough.^20^ Fourth, we should not expect the views that emerge from a deliberative process to reflect the pre‐deliberative views of the population. Deliberation can change people's preferences.^21^ Therefore, whatever the composition of the group, the views that emerge from a deliberative group may not align with (non‐deliberated) public opinion. This is not necessarily a problem. At least, those committed to deliberation cannot object to the involvement of bioethicists simply because their opinions differ from those of the general public.

Nonetheless, concerns over lack of representation amongst expert groups, such as bioethicists, often lead to calls for wider (more inclusive) participation in policy decisions. This is usually identified with democratising the process. However, while involving the public might make decisions more democratic in some respects, the various options are also fraught with problems. The following sections consider three ways in which decision‐making might be made more responsive to public preferences: direct referenda, mini‐publics, and elected representation. While these proposals each have some merits, they all suffer from important limitations. In particular, despite their claims to greater inclusiveness, all of them exclude certain affected groups, such as children, future generations, foreigners, and/or non‐human animals.

## 2 Direct Referenda

If we want mass public involvement in policymaking, then one option is to hold a referendum.^22^ This allows all those eligible to vote to have a say. Moreover, the issue can be separated from other issues that are usually bundled together in elections.

A referendum is often supposed to reveal the will of the people, but this assumes that there *is* such a thing to be discovered. This is problematic, for several reasons. First, it may be that many individuals do not have clear, settled views on the topic. Second, even if each individual has a clear preference, there may not be any coherent *collective* preference.^23^ Third, even if there is a determinate will of the people, a referendum may not discover it. The choice facing voters is usually a crude yes or no, which might not allow them to express what they really want or find a compromise.

Further, if turnout is low, a majority of votes might not reflect the preferences of wider population.^24^ This is especially likely when turnout is unequal.^25^ This might not be problematic, if it gives those more affected a greater say.^26^ However, this assumes that turnout correlates with affectedness. But strength of subjective feeling need not correspond to their objective interests. Thus, participatory processes need not empower the most affected. Rather, certain groups may be more likely to participate for other reasons, such as social capital or free time. Thus, systems of voluntary mass participation may unwittingly privilege such groups, even if they are not the most affected. [32, p. 234; 33, p. 25] While these problems also apply to electoral turnout, participatory inequalities may be greater in referenda. First, more frequent voting may lead to ‘voter fatigue.’ [34] Second, if the issue crosses party lines, it may be harder for voters to decide how to vote [35, p. 112]

More significantly, even if public opinion can be identified, this might not be a good way to settle bioethical questions. The public is often uninformed—or even *mis*informed—about matters, especially involving new technologies. This is unsurprising, since they have little incentive to be informed. Uninformed public opinion may be contrary to scientific evidence and/or unfair to minorities or disenfranchised groups [36].

## 3 Mini‐Publics

Involving the whole public is impractical. Hence, modern democratic states are primarily *representative* democracies. The people do not rule directly. Rather, others rule on their behalf. But those who exercise power are supposed to represent those they rule over. Thus, the people supposedly retain ultimate control, though their power is exercised only indirectly.

Representation can take different forms. Probably most familiar is electoral representation, which is discussed later. However, there is another option, which is arguably closer to the ideal of mass popular participation. One could use random selection (sortition) to appoint a group of ordinary people to represent the wider population [37, 38]. Numerous proposals have been developed, with the numbers selected ranging from around ten to hundreds. These are referred to by various names, such as juries, panels, and assemblies. I will use the term ‘mini‐publics’ here to cover all such proposals. While there are important differences between these proposals, most of my comments are intended to apply to all (or most) of them.

Some advocates of mini‐publics aspire to radical transformation of democratic communities [39]. Most, however, aim to supplement—rather than entirely replace—electoral representation. For instance, in a bicameral system, one chamber might be elected and the other chosen randomly [40]. Alternatively, temporary mini‐publics can be formed to consider specific policy issues, such as abortion or end‐of‐life care. Elected leaders might choose to employ such a mini‐public as an alternative to deciding the matter themselves or calling a referendum.

Whatever the details, sortition has various attractions. Some advocates hope that it will reduce the dominance of professional politicians and political parties [41]. Decisions would be more likely to reflect what ‘ordinary people’ think. However, since decisions would be made by a small group, there would be more opportunity for deliberation than in a referendum. Those selected might be better informed and, perhaps, change their mind on the issue [42]. Thus, the conclusions of a mini‐public need not align with the non‐deliberative views of the wider public. Nonetheless, their decisions might be what the wider population *would* have decided, had they also been through the same deliberative process.^27^

The legitimacy of mini‐publics rests on their representativeness [43, p. 70] Or, more specifically, it rests upon a particular—descriptive—understanding of representation. The mini‐public ideally represents the wider population in virtue of sharing similar characteristics (age, sex, race, religion, etc). Critics sometimes object that descriptive representation is not what really matters in politics [44, ch. 4] Rather, what matters is whether decisions take into account the perspectives and interests of those represented. This is often called ‘substantive representation’ [44, ch. 10]

In principle, someone can represent (substantively) others with whom they have little in common. However, while descriptive representation may not be *necessary* for substantive representation, it can nonetheless be *conducive* to it [45, 46]. A representative from the same group background is likely to share certain experiences and perspectives, making it easier for them to understand and communicate with those they represent. Indeed, it might even make communication unnecessary. If the representative's own views and interests are the same as those of their constituents, then there is less need to worry about accountability or control, since their actions will naturally align with what their constituents want.^28^

Despite its advantages, sortition also suffers from certain problems. These do not necessarily disqualify it from playing some part in wider democratic systems, but they do suggest that it might be unwise to rely extensively on sortition. I will focus on two potential problems. First, the danger that sortition will lead to bad decisions and, second, worries that in practice it will not be as representative as hoped.

Regarding the first, there are various reasons why mini‐publics might lead to inferior decisions. One is that the members of a mini‐public (especially a single‐issue one) might lack an appreciation of wider context and the need for joined‐up policymaking. Even if their decision made sense considered in isolation, it might not cohere well with other policies and decisions. Moreover, there are questions about the decision‐making competence of ordinary people. Lotteries do not select for (or against) any particular qualities, such as wisdom or experience. Those selected need not be best qualified, or even minimally qualified, for the role. Even if mini‐public members are capable of learning on the job, they would likely be reliant on others (civil servants, expert advisers, or the like) during this learning phase. This might give disproportionate influence to those in these (unelected and usually unaccountable) advisory positions, rather than the representative mini‐public. This potentially undermines the supposed superiority of a mini‐public, as a way of giving power to ordinary citizens rather than experts or other elites.^29^

The second problem is that a mini‐public might not be as representative, in practice, as suggested in theory.^30^ Most authors propose that service should be voluntary (usually compensated, but not mandatory).^31^ However, not everyone selected would be willing or able to serve in such mini‐publics. Some groups will be more likely to accept a summons than others, either due to their interest in the topic or because they have more spare time (e.g., the retired or unemployed). This could skew the representativeness of the mini‐public. In a fully random approach, individuals are not selected for any particular property or characteristic. Therefore, it is unclear how a replacement should be chosen.

One possibility is to use a form of stratified sampling, rather than a fully random approach.^32^ Then, when someone refuses, a replacement could be drawn from the same group.^33^ However, this is more complicated that it sounds. First, there is some difficulty identifying relevant characteristics.^34^ To illustrate, suppose we begin with quotas for men and women.^35^ Then, if a woman declines to serve, another woman can be called up as her replacement. But, if white women are more likely to accept than black women, this could still lead to under‐representation of black women (and black people more generally). That could be solved by further stratification, for instance selecting quotas from four groups (white men, black men, white women, black women). Now, if a black woman declines an invitation, another black woman can be selected to replace her. But there are still differences within this group. Suppose someone selected is a young, black, Muslim woman who is a single parent and a college drop‐out. A replacement is unlikely to be all of these things, unless we have a large number of very specific quotas. Furthermore, even if quotas can ensure representation of certain salient groups, they run contrary to the logic of random selection and potentially undermine the idea that all should have equal chances of selection [52]. So long as service is not compulsory, it is always possible that there is a significant difference between those who are willing to serve and those who are not.

Moreover, even if the membership of the mini‐public is broadly representative, some members may be more effective advocates than others. Deliberative democrats typically prioritise a certain style of reasoned discussion, which may be more hospitable to privileged groups and tends to exclude those (seen as) too emotional, partisan and so forth [53, 54]. Thus, those who are educated, confident, and native speakers might dominate discussion, leaving other groups under‐represented (even if present).^36^ One potential remedy is to permit a wider range of deliberative contributions, such as allowing more emotional speech. However, this can also be exclusionary. Those who find the emotions uncomfortable and hard to overcome may withdraw from discussions. This can also favour those with higher social status [55, pp. 767–68] Moreover, even when members of marginalised groups do speak, their views may not be given a fair hearing by others [56, 57]. Again, it is unclear whether mini‐publics really live up to their representative aspirations.

Moreover, mini‐publics can only include, and thus represent, those who are eligible for selection. Even if the members represent the candidate pool from which they are drawn, they are not going to represent other groups, such as young children, future generations, or (usually) foreigners. Thus, the interests of these excluded groups may still be neglected.

## 4 Electoral Representation

Representative institutions are usually composed of elected politicians, rather than being selected at random. These elected politicians are often descriptively unrepresentative of those they are supposed to represent. However, the fact that they owe their position to election is supposed to ensure their accountability and responsiveness to their constituents, and thus substantive representation. If they are not sufficiently responsive, then they risk being voted out and replaced. Thus, while electoral representation may be farther from the ideal of popular rule than either referenda or mini‐publics, electoral accountability may make it more defensible than other forms of elite rule [58]. So, lack of descriptive representation amongst elected officials may be less problematic than a similar lack in unelected groups, such as bioethicists.

However, while elections can preserve some element of responsiveness to the people, they are limited in what they can achieve. A vote in an election may convey various things. It may be a retrospective judgement of how well (the voter thinks) the present incumbent has served over their past term and/or a prospective judgement about who will do best in the future. Moreover, it will, ordinarily, be based on a number of different issues. Thus, we cannot assume that voters endorse all of the policies of the candidates that they vote for. They might vote for someone *despite*, rather than because of, a particular policy commitment. And, depending on the voting system, they might also be forced to choose between supporting a candidate and a party. Again, it is difficult to infer their underlying preferences from their vote.

Electoral systems also affect representative accountability. In non‐proportional ‘Westminster’ systems, voters typically choose a single representative for their district or constituency. These systems can give voters little choice, since they tend to produce a smaller number of parties competing for the middle ground. Voters might be forced to vote for ‘lesser evil’ candidates.^37^ Further, the distribution of seats may differ significantly from the distribution of votes.^38^ On the other hand, proportional representation (PR) produces a better correspondence between popular vote and parliamentary representation. However, this also threatens accountability [60]. A party may win a significant share of the seats, yet still be excluded from a governing coalition. Conversely, smaller parties can sometimes exert disproportionate influence, especially if positioned as ‘kingmakers’. And, since government policy is often down to coalition agreements, it is difficult to identify who is responsible. For these reasons, it is difficult for voters to ‘throw the rascals out’ if dissatisfied.

Finally, as with referenda, electoral representation can be distorted by unequal turnout. Representatives may ignore groups that are less likely to vote. Again, the problem is even worse for disenfranchised groups, such as children and future generations. If representatives focus on re‐election, these groups are likely to be neglected.

## 5 A Role for Bioethicists

Calls to democratise bioethics, by involving ordinary citizens or their elected representatives, may be a response to the lack of descriptive representation among bioethicists (§1). However, various proposals for democratisation—including popular referenda, mini‐publics, and elected representation—are beset with their own problems. Even if they empower certain groups to advocate and protect their own interests, those included are necessarily limited. Other groups, such as young children or future generations, will always be excluded; they cannot speak for themselves.

I suggest that bioethicists are often well placed to speak out against policies that might be harmful. I do not make the stronger claim that they are uniquely, or even best, placed to do this. There may well be others who can do a similar job.^39^ Nor do I suggest that bioethicists have privileged access to moral truths. Nonetheless, bioethicists can serve several useful functions when deliberating on policy proposals. For one example, they can identify potential implications of proposals, which might help to identify valid (though not necessarily sound) arguments for or against them.^40^ Bioethicists are also accustomed to looking at issues from different perspectives and raising objections, including potentially unpopular ones, both to new proposals and to existing practices. And their conceptual innovations sometimes produce new ways of viewing familiar issues.^41^

While there is indeed a worry that the lack of descriptive representation amongst bioethicists may lead them to neglect certain problems, this is less serious when their role is primarily a negative one (raising objections, rather than making policy). First, the objections that they raise do not have to be made on their own behalf. They may raise objections on behalf of others, who may not be able to speak for themselves. Second, the costs of failure are lower here. Suppose bioethicists fail to raise an objection to a certain policy, because they fail to consider some unrepresented perspective. This does not prevent others from raising the objection.

Even if the role of bioethicists is primarily negative, it might still be objected that it is undemocratic to entrust them with any special power or influence. However, I contend that this is not necessarily undemocratic or, at least, not in any sense that makes it objectionable. Democracy, as usually practised, routinely involves certain departures from popular rule, particularly to protect minority groups. Even representation, whether by lottery or election, introduces an inequality between representatives and represented. This suggests that democracy should not simply be identified with popular rule.^42^

In fact, there is a long tradition emphasising the importance of checks and balances, to prevent popular tyranny. The idea of a mixed constitution can be traced back at least to Aristotle, who suggested measures to balance the power of rich and poor [65, book VI, ch. 3] Admittedly, Aristotle did not call this mixed regime a *democracy*—he used that word for an inferior constitution dominated by the poor. Today, what Aristotle would call democracy might instead be referred to as populism or ochlocracy (mob‐rule). The regimes we regard as democratic (in a commendatory sense) are often mixed regimes, combining popular elements with checks and balances.

Those who defend democracy today often have in mind a mixed regime, rather than pure popular rule. Bernard Crick, for instance, rejects the populist pursuit of a unified general will, holding that politics requires diversity [66]. Anthony McGann argues that the instability of majority preferences, far from undermining popular rule, is actually beneficial in ensuring rotation of winners and losers [67]. Philip Pettit suggests that the crucial function of democratic institutions is not that the people author the laws, but that they can contest policies contrary to their interests [68]. Similarly, Samuel Bagg contends that the goal of democracy is preventing state capture [69]. Despite important differences, these various thinkers all agree that democracy is not—or should not be—simply about translating the will of a numerical majority into policy. Even majority power sometimes needs to be limited to protect minorities.

Representation is one mechanism for restraining majority power, at least if representatives have some freedom to resist popular impulses. Even elected representatives may do this to some degree. However, representation can also take other forms, which need not depend on either descriptive similarity or electoral accountability. For instance, activist groups such as Greenpeace, Oxfam, and PETA may represent (speak for) the interests of the environment, global poor, and non‐human animals respectively. These representative claims have various bases, besides elections or similarity, such as tradition, hypothetical consent, special expertise, or giving voice to interests that would otherwise be neglected [70, 71]. In some cases, it may be the *lack* of electoral accountability that enables these representatives to oppose a popular majority. Admittedly, these representative claims may be controversial, particularly when they seem to subvert the agency of groups who could otherwise speak for themselves. However, even imperfect representation may be better than exclusion. Moreover, this may be the best that can be hoped for, when it comes to groups who cannot speak for themselves (such as future generations).

Another protective mechanism, found in many (though not all) democracies is judicial review. Here, judges may be empowered to strike down legislation that violates constitutional rights. Some argue that this practice is undemocratic, precisely because it is anti‐participatory, anti‐egalitarian, and anti‐majoritarian.^43^ However, this again rests on a narrowly procedural understanding of democracy. Judicial activism can promote democratic values, such as respect for rights [74, 75]. While empowering judges is contrary to the democratic ideals of political equality and participation, this is also true of representation [76]. Further, Annabelle Lever argues that, despite being unelected, judges can embody democratic forms of representation, accountability, and participation [77]. Perhaps bioethicists (or other scientific experts) could be subject to similar informal accountability.^44^

It is beyond the scope of this paper to provide, let alone defend, a full theory of democracy. However, my aim has been to show that there is more to democracy that increasing public participation or the control of the majority. That is not to say that there is no place for these things, but there is sometimes reason to resist or restrain the popular will. Moreover, this is not necessarily to compromise democracy for the sake of something else, but may be part of a richer understanding of democracy.

## 6 Conclusion

Democratic decision‐making in bioethics and health policy can mean many things. It might mean citizens making decisions directly, through a referendum, or indirectly, through representatives (whether randomly selected or elected). All of these methods may have some place, but they are not all that there is to democracy. Democracy is not simply about realising whatever (a majority of) the people want. Mechanisms such as judicial review and, indeed, representation itself are departures from equal participation. Nonetheless, they can serve democratic ends. Something similar may be true when bioethicists are involved in deliberations on policy. While their special influence is a departure from equal public inputs, it can help to prevent democracy from degenerating into a tyranny of the enfranchised majority.

## Conflicts of Interest

The author declares no conflicts of interest.

## Data Availability

The author has nothing to report.

## Endnotes

1 I do not mean to suggest that morality has no bearing on these questions. Someone who thinks a certain practice, such as abortion, morally wrong is likely to oppose it being permitted, funded and so forth. However, my point is that these decisions are made *for* a whole political community, rather than a single individual or family. This leads many to conclude that these decisions should be made *by* the whole political community.

2 See, for example, [1, 2, 3].

3 Of course, democracy is not the only political system, but it is widely regarded as the most legitimate. For illustrative calls for democratic decision‐making, see for example, [4, 5].

4 I take no stance here on who counts as a bioethicist. It might include those from various backgrounds (law, medicine, philosophy, social science) and employed in universities, hospitals, NGOs and so forth. How my suggestions are best implemented may depend on local context.

5 Epistocracy can take various forms [6, p. 175], such as plural voting [7] or restricted suffrage [8].

7 One lesson that the authors draw [15, p. 19] is that steps should be taken to diversify bioethics. I have no objection to this. However, I think bioethicists can still play a legitimate role in influencing policy despite being descriptively unrepresentative. (Note also that bioethicists can never be like the whole population in *all* respects, for example, education or occupation.)

8 I mean they should have *direct* input, as opposed to indirect influence through shaping public opinion.

9 On presidential commissions in the USA, see [17].

10 I thank an anonymous referee for suggesting this alternative.

11 Indeed, even representation involves some departure from equal inclusion. However, representatives are supposed to act on behalf of those they represent. This responsiveness can be facilitated by random selection or electoral accountability.

12 Descriptive representation is sometimes referred to as statistical or ‘mirror’ representation.

13 Here ‘foreigners’ includes both resident non‐citizens (immigrants) and those residing overseas, who might nonetheless be affected by certain decisions.

14 For instance [19], suggests that economic policy should be left to economists rather than politicians, but that a similar approach would be undesirable for policies with a moral dimension, such as abortion (p. 119).

15 A recent survey of US bioethicists found that they tend to be more white, male, and non‐religious than the population as a whole [15].

16 For illustration of competing visions of the field, see [21].

17 For instance, ‘Christian bioethics’ and ‘feminist bioethics’ are both well‐established sub‐fields and, more recently, bioethicists have drawn on many non‐Western traditions, such as Confucianism [22], Islam [23], and African philosophies [24].

18 More accurately, this does not tell us about *all* bioethicists, only those who were eligible for and responded to the survey. But this does not affect the point about selective recruitment to redress imbalance.

19 One could similarly select a panel with certain viewpoints. It might be objected that this selection biases their deliberations, although there may sometimes be reasons for this.

20 The need for proportional numbers is rejected by [18, p. 524] and [25, p. 484].

21 Preferences are not taken as given, but (trans)formed through deliberation [26, 27].

22 Not all advocates of public input go this far. Some merely think that decision‐makers should be informed of public opinion. For example [28, p. 338].

23 Even in a simple case, with three options and three voters, it might be that a majority prefer A to B, a majority prefer B to C, and yet a majority prefer C to A.

24 Suppose 60% vote in favour of the proposal, on 50% turnout. The proportion of the whole population in favour could be anywhere between 30% and 80%.

25 Unequal turnout is sometimes seen as threatening political equality, for example [29].

26 See [30], drawing on [31].

27 For such a suggestion, see [27, p. 43; 43, p. 71]. This assumes there is *one* thing that the people would think. However, if individuals can reach different conclusions, having undergone the same deliberative process, then different groups of people might also reach different conclusions. Perhaps we should say only that a particular poll outcome represents one possible conclusion that the whole group *might* have reached. They might, however, have arrived at another decision.

28 This is sometimes called ‘gyroscopic’ representation [47, p. 520].

29 An anonymous reviewer asks whether this point is in tension my defence of a role for bioethicists. However, my objection is not to expert influence. My point is that mini‐publics fail to provide a genuine alternative to it. Moreover, they may be worse, if they make expert influence less transparent and accountable.

30 For an informative discussion of difficulties in one high‐profile case, see [48].

31 Voluntariness is proposed by [38, p. 457; 49, p. 156; 20, p. 799]. For an exception, proposing mandatory service, see [50, pp. 370–371].

32 Guerrero mentions such a possibility, but expresses a preference for pure lotteries [49, p. 167, n.31].

33 As suggested by [38, p. 459].

34 Memorably put in [51, p. 437].

35 I set aside the difficulties of dealing with trans and non‐binary individuals, though these demonstrate that even this ‘simple’ example may not be that simple.

36 Again, my objection is not to giving more influence to the educated. Rather, my aim is to rebut objections to this, from defenders of mini‐publics, by showing that they may do the same thing anyway.

37 This problem might be mitigated by preferential voting [59].

38 If every constituency was divided 55% Red and 45% Blue, the Reds would win all seats, while the Blues would have no legislative representation.

39 An anonymous reviewer asked whether the argument is distinctive to bioethicists or would extend to other expert groups. My focus here is on bioethicists, so I do not address how far the argument might generalise to other experts. However, I would not be troubled if it did.

40 This is what [61] terms ‘weak expertise’.

41 For examples of bioethicists challenging conventional concepts, see [62, 63, 64].

42 Alternatively, we may say that democracy is simply popular rule, but this is not all that we value. In this case, democracy may be limited for the sake of other ends, such as justice. Thus, what we want is not simply democracy but rather a liberal or just democracy.

43 Probably the most prominent critic is Jeremy Waldron, for example [72], though this worry is even expressed by some US Supreme Court Justices [73, p. 8].

44 This accountability might be enhanced by greater transparency over disagreements. For instance, scientific advisers might emulate Supreme Court justices by writing dissenting opinions, as proposed by [78, p. 570]. Some bioethics bodies operate in this manner [1, p. 329].
